# The Biogeographical Distribution of Soil Bacterial Communities in the Loess Plateau as Revealed by High-Throughput Sequencing

**DOI:** 10.3389/fmicb.2018.02456

**Published:** 2018-10-18

**Authors:** Dong Liu, Yang Yang, Shaoshan An, Honglei Wang, Ying Wang

**Affiliations:** ^1^State Key Laboratory of Soil Erosion and Dryland Farming on the Loess Plateau, Northwest A&F University, Yangling, China; ^2^Kunming Institute of Botany, Chinese Academy of Sciences, Kunming, China

**Keywords:** Chinese Loess Plateau, soil pH, 16S rRNA high-throughput sequencing, beta diversity, longitudinal gradients

## Abstract

The rigorous environmental stress of the severely eroded Loess Plateau may have promoted specific soil bacterial communities in comparison to other eco-environmental regions. In order to unmask the bacterial diversity and most influential environmental parameters, Illumina MiSeq high throughput sequencing of 16S rRNA from 24 representative soil samples collected across south-east to north-west transect of the Loess Plateau in northern Shaanxi, China was conducted. This high-throughput sequencing revealed a total of 1,411,001 high quality sequences that classified into 38 phyla, 127 classes, >240 orders, and over 650 genera, suggesting a high bacterial richness across the Loess Plateau soils. The seven dominant groups were: *Proteobacteria, Actinobacteria, Acidobacteria, Planctomycetes, Gemmatimonadetes, Chloroflexi*, and *Verrucomicrobi* (relative abundance of >5%). Increasing/decreasing soil pH and geographic longitudinal distance correlated significantly with increasing/decreasing bacterial richness and diversity indices. Pairwise correlation analysis showed higher bacterial diversity at longitudinal gradients across 107°39′-109°15′ (south-east to north-west) in our studied Chinese loess zone. Variation partitioning analysis indicated significant influence of soil characteristics (~40.4%) than geographical distance (at a landscape scale of ~400 km) that was responsible for 13.6% of variation in bacterial community structure from these soils. Overall, contemporary soil characteristics structure the bacterial community in Loess Plateau soil to a greater extent than the spatial distances along the loess transect.

## Introduction

Soil microorganisms from terrestrial habitats are key drivers of critical ecosystem services, such as nutrients cycling (Madsen, 2005), soil organic matter (SOM) stabilization and C sequestration (Grandy and Neff, 2008; Plaza et al., 2013). Although, recent investigations (Xiong et al., 2012; Liu et al., 2015, 2018; Xia et al., 2016) across different ecosystems have exhibited distinct biogeographic distribution pattern of soil microbes, deeper insights into their distribution and relationship between microbial-mediated nutrient cycling is required to facilitate our understanding of such processes. In recent decades, developments in next-generation high-throughput sequencing have made comprehensive study of soil microbial communities affordable at various scales (Kuang et al., 2013; Wu et al., 2013; Liu J. et al., 2014; Zhang et al., 2018).

In comparison to the European and American counterparts, comprehensive information on biogeographic distribution of soil bacterial communities in Chinese soils is limited. Recently, some efforts in gathering this information were made in mono-ecosystems of northern and northeast part of China (Liu J. et al., 2014; Wang et al., 2015). The Chinese Loess Plateau spans across a total area of 6.4 × 10^5^ km^2^ and is widely considered as one of the most severely eroded regions in the world, making it a target place for implementation of Grain to Green Program (GTGP) (Deng et al., 2015). With the country's ambitious “payment for ecosystem services” (such as soil carbon storage, biodiversity conservation and climate regulation) program (Bennett, 2008), the Chinese government has invested ~28 billion USD in GTGP during its first implementation period from 1999 to 2008 (Lü et al., 2012). This substantially resulted in land-use conversions from former agricultural lands to re-vegetating forests and/or natural-recovering grasslands (Deng et al., 2014; Fazhu et al., 2015). The implementation of GTGP has greatly improved soil carbon concentrations, yet the soils in Loess Plateau are still characterized with deficient total phosphorus and nitrogen contents (Liu et al., 2013). There have been assessments on soil nutrients and land use shifts (Fu et al., 2000; Zhang et al., 2010; Fazhu et al., 2015), landscape positioning (Wang et al., 2001), the relationship between soil properties and microbial diversity (Yang et al., 2018) as well as changes in soil bacterial communities along a vegetation gradient (Zeng et al., 2016). However, biogeographic distribution patterns of bacterial communities across Loess Plateau soils still lack in-depth exploration.

In the loess plateau region, previous studies have made efforts in revealing microbial communities structures. However, they mainly focused on soil microbes in mono-ecosystems, such as grasslands (Huhe et al., 2017) or agricultural lands (Tian et al., 2017). Most recently, Yang et al. (2018) investigated 5 land use types to test the relationship between microbial diversity and soil carbon storage, but the study was still not representative of microbial patterns in this region as the soils were collected from a river catchment. Soil microbes exhibit spatial aggregation instead of random distribution (Martiny et al., 2006; Katsalirou et al., 2010), which is generally determined by historical factor of dispersal limitation (i.e., geographic distance) and contemporary environmental heterogeneity (Fierer, 2008; Ge et al., 2008; Kuang et al., 2013). These two driving factors shaping microbial communities are scale-limited and debatable. The popular dictum “everything is everywhere, but the environment selects” (de Wit and Bouvier, 2006) indicates the control of external factors regarding environmental variations as strong factors regulating bacterial biogeographical persistence (Fierer and Jackson, 2006; Fuhrman et al., 2008; Schauer et al., 2010). Among series of soil parameters, such as SOC, total nitrogen (TN) and availability of micro-nutrients (Fierer and Jackson, 2006; Rousk et al., 2009; Xiong et al., 2012; Liu J. et al., 2014), soil pH is considered as a prime factor shaping bacterial community structure in soils. Apart from the contemporary environment, a significant effect of geographic distance has also been proposed in some large scale studies (Ramette and Tiedje, 2007; Ge et al., 2008; Schauer et al., 2010). However, at the moderate/landscape scale (~500 km), it is unclear whether geographic distance, relative to local environment, is an equally important and/or dominating factor toward distribution of soil microbial communities.

In this study, we collected 24 soil samples from south-east to north-west transect in the hilly area of loess plateau of northern Shaanxi province in China. The area is covered with vegetation ranging from forest, grassland, and agricultural lands at different landscape scales (Figure S1). Spatial variations in bacterial community structure from these soils were investigated by 16S rRNA (bacterial biomarker) gene sequencing (V4 hypervariable region of the gene) on Illumina MiSeq high-throughput sequencing platform. The objectives of this study were: (1) to determine the diversity and structure of bacterial communities across Loess Plateau soils; (2) to examine the dominant factors in structuring the distribution of the soil bacterial community at such a landscape range; (2) to explore the biogeographic patterns of soil bacterial communities of these soils.

## Materials and methods

### Site description and soil sampling

The study region is located in the southeast to northwest (~400 km) hilly transect of loess plateau in northern Shaanxi province of China (107°39′-109°15′E, 34°4′-37°31′N, altitude: 415–1,633 m a.s.l.). The region is characterized by semi-arid continental climate with an average annual rainfall of 513 mm. The mean annual air temperature is ~8.8°C. Soils in the study area are mainly derived from loess, according to the soil classification system of the Food and Agriculture Organization of the United Nations (FAO) and further classified as Calcic Cambisols (IUSS Working group WRB, 2014) with silty loam texture. A transitional environment from south to north is represented by forest, forest-grassland, and grassland to dessert grassland vegetation belts. Qingling (QL) and Ziwuling (ZWL) represent locations of mountain forests; Liandaowan (LDW) represents sites from grassland township; Guanzhong (GZ), Luanchuan (LC) and Weibei (WB) refer to agricultural areas from three Loess tablelands, respectively (Figure S1); while Ansai county (AS) and Jingbian county (JB) from regions of forest steppes (Figure S1). The codes of A, B and C following the above-mentioned site abbreviations represent various biological replicates at landscape scales. The geographical and typical vegetation characteristics of the investigated sites are as given in Table 1. In each site, three replicate subplots were randomly selected with an area of 15 m × 15 m and six replicate soil subsamples were collected from each subplot and at the soil depths of 0–10 cm using auger boring with a drill size of 20 cm length and 2 cm diameter. 6 subsamples of identical soil depths were sieved through a 2 mm mesh and then mixed to form a composite sample of ~100 g. A 10 g subsample from each plot was immediately wrapped in aluminum foil, quenched with liquid N_2_, and stored at −80°C laboratory conditions until the extraction of soil DNA.

**Table 1:** Site geographic information and soil physicochemical parameters used for this study.

**Sample**	**Location**	**Vegetation**	**Latitude (N)**	**Longitude (E)**	**Altitude (m asl)**	**Slope (°)**	**pH**	**Soil organic C (g kg^−1^)**	**Total N (g kg^−1^)**	**Total P (g kg^−1^)**	**Moisture (%)**	**Number of phylotype^a^**	**Phylogenetic diversity^b^**
QL-A	Qingling mountain	*Quercus wutaishanica*	34°6′25.74″	108°11′36.7″	708	0–15	6.80	15.03	1.28	0.56	16.1	2,710	164.2
QL-B	Qingling mountain	*Quercus acutidentata*	34°10′52.14″	107°39′2.15″	754	42	7.65	21.63	1.99	0.59	17.6	3,968	255.2
QL-C	Qingling mountain	*Quercus wutaishanica*	34°4′15.23″	108°1′5.27″	822	10–30	6.14	14.87	1.31	0.54	12.8	3,036	183.4
GZ-A	Guanzhong, Qishan	*Zea mays L*.	34°25′59.98″	107°39′45.96″	656	1–5	8.54	9.89	1.11	0.93	17.2	4,128	243.4
GZ-B	Guanzhong, Fufeng	*Zea mays L*.	34°20′28.46″	108°0′41.66″	549	1–5	8.43	9.95	1.02	0.95	13.8	3,899	237.0
GZ-C	Guanzhong, Xingpin	*Zea mays L*.	34°18′15.43″	108°22′53.05″	415	0–5	8.55	12.49	0.94	1.18	15.2	3,964	247.1
WB-A	Weibei, Fuping	*Amygdalus persica L*.	34°43′55.22″	109°13′45.74″	421	0–5	7.81	10.00	1.01	1.1	7.2	4,694	296.2
WB-B	Weibei, Pucheng	*Pyrus spp*	34°54′38.12″	109°36′4.50″	397	0–5	8.42	9.92	1.00	1.38	12.5	4,099	255.8
WB-C	Weibei, Changwu	*Malus pumila Mill*.	35°11′45.60″	107°48′26.13″	1,180	0–5	7.78	9.06	1.20	1.52	19.5	3,285	209.4
LC-A	Luochuan county	*Malus pumila Mill*.	35°47′43.77″	109°28′31.76″	1,206	0–5	8.32	8.09	0.97	0.96	16.5	4,550	281.5
LC-B	Luochuan county	*Malus pumila Mill*.	35°49′30.47″	109°29′0.83″	1,220	1–3	8.31	8.61	1.09	1.05	11.2	4,359	262.1
LC-C	Luochuan county	*Malus pumila Mill*.	35°50′32.16″	109°24′28.55″	1,138	1–3	8.45	6.88	0.77	0.68	12.7	4,732	300.4
ZWL-A	Ziwuling mountain	*Pinus tabuliformis*	36°0′18.15″	109°5′54.50″	1,185	30–40	8.38	15.62	1.36	0.54	14.4	3,654	231.7
ZWL-B	Ziwuling mountain	*Carrière*	36°1′42″	108°52′43″	1,025	0–15	8.87	10.59	0.95	0.47	18.5	3,786	239.2
ZWL-C	Ziwuling mountain	*Betula platyphylla*	36°5′56.70″	108°37′28.56″	1,151	30–45	8.73	18.76	1.41	0.58	14.5	3,631	205.7
AS-A	Ansai county, fangta	*Artemisia gmelinii*	36°47′23.30″	109°16′32.67″	1,273	21	8.9	2.58	0.32	0.53	13.6	4,294	252.5
AS-B	Ansai, zhifanggou	*Caragana Korshinskii*	36°43′54.22″	109°15′29.74″	1,298	10	8.96	2.69	0.33	0.49	8.1	4,065	238.5
AS-C	Ansai, zhifanggou	*Robinia pseudoacacia*	36°43′56.05″	109°15′18.10″	1,335	17	8.79	4.00	0.47	0.56	15.6	3,897	266.4
LDW-A	Liandaowan	*Bothriochloa ischaemum (L.)*	37°11′39″	108°58′15″	1,350	30–40	8.82	5.05	0.45	0.46	18.9	3,790	231.5
LDW-B	Liandaowan	*Artemisia scoparia Waldst*.	37°10′12″	108°57′01″	1,420	0–5	9.11	2.66	0.18	0.47	13.8	4,220	247.6
LDW-C	Liandaowan	*Stipa grandis*	37°11′49.06″	108°57′11.34″	1,403	7–20	9.12	4.09	0.27	0.49	14.6	4,251	252.0
JB-A	Jingbian county	*Salix cheilophila*	37°31′20.21″	108°51′38.41″	1,563	0–5	8.91	2.91	0.27	0.32	11.2	3,699	266.6
JB-B	Jingbian county	*Salix matsudana Koidz*.	37°30′9.63″	108°52′58.06″	1,633	1–10	9.02	2.61	0.29	0.26	7.6	4,087	246.7
JB-C	Jingbian county	*Populus simonii Carr*	37°28′16.91″	108°54′8.08″	1,572	1–10	8.92	4.54	0.45	0.43	9.5	4,407	260.6

a *The number of phylotype was calculated from 8,000 sequences per soil sample at the 97% similarity lever*.

b *These values (PD, whole tree) were calculated using Faith's index*.

### Soil physicochemical properties

Soil water content was measured gravimetrically by oven drying moist soil samples (105°C, 24 h). Soil pH was determined in a soil-to-water (1:2.5, W/V) mixtures of dry soil and distilled water using a Delta 320 pH meter (Mettler-Toledo Instruments (Shanghai, China) Co., Ltd). Soil organic carbon (SOC) was determined via wet oxidation using dichromate in an acid medium followed by the FeSO_4_ titration method (Bao, 2007). Total nitrogen (TN) contents were measured with Kjeldahl digestion and distillation azotometry following the extraction with 0.02 mol/L sulfuric acid (Reed and Martens, 1996). Total phosphorus (TP) was measured spectrophotometerically after wet digestion with H_2_SO_4_ and HClO_4_ followed by colorimetric analysis (UV 2800; Parkinson and Allen, 1975).

### DNA extraction and PCR amplification

Soil DNA was extracted from 0.2 g soil samples using the MoBioPower Soil DNA isolation kit (12888) following the manufacturer's instructions. The extracted soil DNA was stored at −20°C until further processing. The V4 hypervariable region of the bacterial 16S rRNA gene was amplified using the forward primers 502F (5′-AYTGGGYDTAAAGNG-3′) and the reverse primer 802R (5′-TACNVGGGTATCTAATCC-3′) (RDP's sequencing Pipeline: http://pyro.cme.msu.edu/pyro/help.jsp). PCR reactions were performed in triplicate in a 25 μL mixture containing 0.25 μL Q5 high-fidelity DNA polymerase, 5 μL of 5X Reaction buffer, 5 μL of 5X High GC buffer, 0.5 μL of dNTP mix (10 mM strength), 1 μL of template DNA (~10 ng), 1 μL of each primer (10 μM) and 11.25 μL of DNase free water. The following thermal program was used for amplification: 98°C for 30 s, followed by 25–27 cycles at 98°C for 15 s, 50°C for 30 s, with a final extension step at 72°C for 5.5 min. The PCR products were quantified on 2% agarose gel. Marker used was DL 2000 DNA Marker takara 3427A and the obtained DNA ladder sizes were 100, 250, 500, 750, 1,000, and 2,000 bp.

### Sample preparation for illumina MiSeq sequencing

Amplicons were extracted from 2% agarose gels and purified using the Axygen Axy Prep DNA Gel Extraction kit (AP-GX-500) following the manufacturer's instructions and quantified on Microplate reader (BioTek, FLx800) using Quant-iT PicoGreen dsDNA Assay Kit, Invitrogen (P7589). Purified amplicons were pooled in equimolar and paired-end sequenced (2×300) using MiSeq Reagent Kit v2 (600-cycles-PE) (MS-102-3003), on an Illumina MiSeq platform (Personalbio, Shanghai) according to the standard protocols.

### Bioinformatic processing of the illumina results

Raw FASTQ files were de-multiplexed and quality-filtered using QIIME (Quantitative Insights Into Microbial Ecology, v1.8.0, http://qiime.org/) with the following criteria: (i) The 300-bp reads were truncated at any site that obtained an average quality score of <20 over a 10-bp sliding window, and the truncated reads shorter than 150 bp were removed and reads containing ambiguous characters were discarded; (ii) the FLASH software (v1.2.7, http://ccb.jhu.edu/software/FLASH/; Magoc and Salzberg, 2011) was used to allign the overlapping sequences that passed the quality screening with ≥10 bp. Mismatched reads were discarded. The remaining sequences were clustered into operational taxonomic units (OTUs) using UCLUST. OTUs with 97% similarity cutoff were clustered on the basis of an open reference by searching reads against the Greengenes database (Release 13.8, http://greengenes.secondgenome.com/; DeSantis et al., 2006), OTUs with the abundance of lower than 0.001% of total amount from the sample sequenced were not considered in final analysis (Bokulich et al., 2012). OTUs assigned to same taxonomy were combined at various taxonomic levels. Phylogenetic diversity of the whole tree (PD, whole tree) was used as the estimation of α-diversity, which incorporates the phylogenetic breadth across taxonomic levels (Faith, 1992; Faith et al., 2009). A cut-off value of 45,000 sequences (per sample) was used for subsequent community analysis, in order to minimize the survey effort (number of sequences analyzed per sample). The differences in the overall bacterial communities between each pair of samples were determined using the UniFrac distance metric analysis (Lozupone and Knight, 2005). This UniFrac analysis is based on estimating branch length of the tree as a measure of phylogenetic distance between the taxonomical groups under question leading to their posterity from either one or the other environment. Both weighted (quantitative) and unweighted (qualitative) variants of UniFrac (Lozupone et al., 2007) are widely used in microbial ecology, where the former accounts for abundance of observed organisms (Lozupone et al., 2007), while the latter only considers their presence or absence (Lozupone and Knight, 2005). The analyses mentioned above were performed using the MOTHUR program (http://www.mothur.org). The primary sequence data is deposited in Sequence Read Archive (SRA) database of NCBI under accession number SRP126984.

### Statistical analysis

The simple linear regression analyses (SPSS 18.0 for Windows) were used to test the relationships between geochemical features and soil bacterial OTUs phylogenetic richness or phylogenetic diversity. For beta-diversity analysis, dissimilarity of bacterial communities was calculated using Pairwise-UniFrac distances between all samples. The effects of soil variables on Beta-diversity of bacterial community structure was determined by correlation analysis based on Mantel's Test (Mantel, 1967) between measured soil variables and OTU dissimilarity matrix. Pairwise UniFrac distances calculated for the total bacterial community analyses were visualized using non-metric multidimensional scaling (NMDS) plots in the R environment. QIIME (Quantitative Insights Into Microbial Ecology, v1.8.0, http://qiime.org/) was used for the cluster analysis (Unweighted pair-group method with arithmetic means,UPGMA) of the bacterial communities based on the NMDS dissimilarity matrix. We conducted canonical correspondence analysis (CCA) to identify the abiotic factors that are significantly related/contributed to soil bacterial communities; these results were used to construct for subsequent variation partioning analysis (VPA) using Canoco 5.0 software to explore, the amount of variability in bacterial communities in response to various abiotic factors (such as geographic distance and other soil physiochemical variables).

## Results

### Soil physicochemical properties

Soil pH ranged from 6.80 to 9.12. Soil organic C and total N varied from 2.58 to 21.63 g kg^−1^ and from 0.18 to 1.99 g kg^−1^, respectively. Pairwise geographic distance (calculated from the coordinates of longitude and latitude) between the sampling regions ranged from 0.29 to 391.22 km (Table S1), respectively. Soil organic C (*r* = −0.527, *P* = 0.008) and total N (*r* = −0.538, *P* = 0.007) showed significant negative relation with geographic distance, and soil organic C and total N contents were higher at lower latitudes than those at higher latitudes (Table 1). In contrast, soil pH was highly positively correlated with latitude (*r* = 0.749, *P* < 0.001) and geographic distance (*r* = 0.506, *P* = 0.012).

### Distribution of bacterial taxa and phylotypes

**Figure 1:**
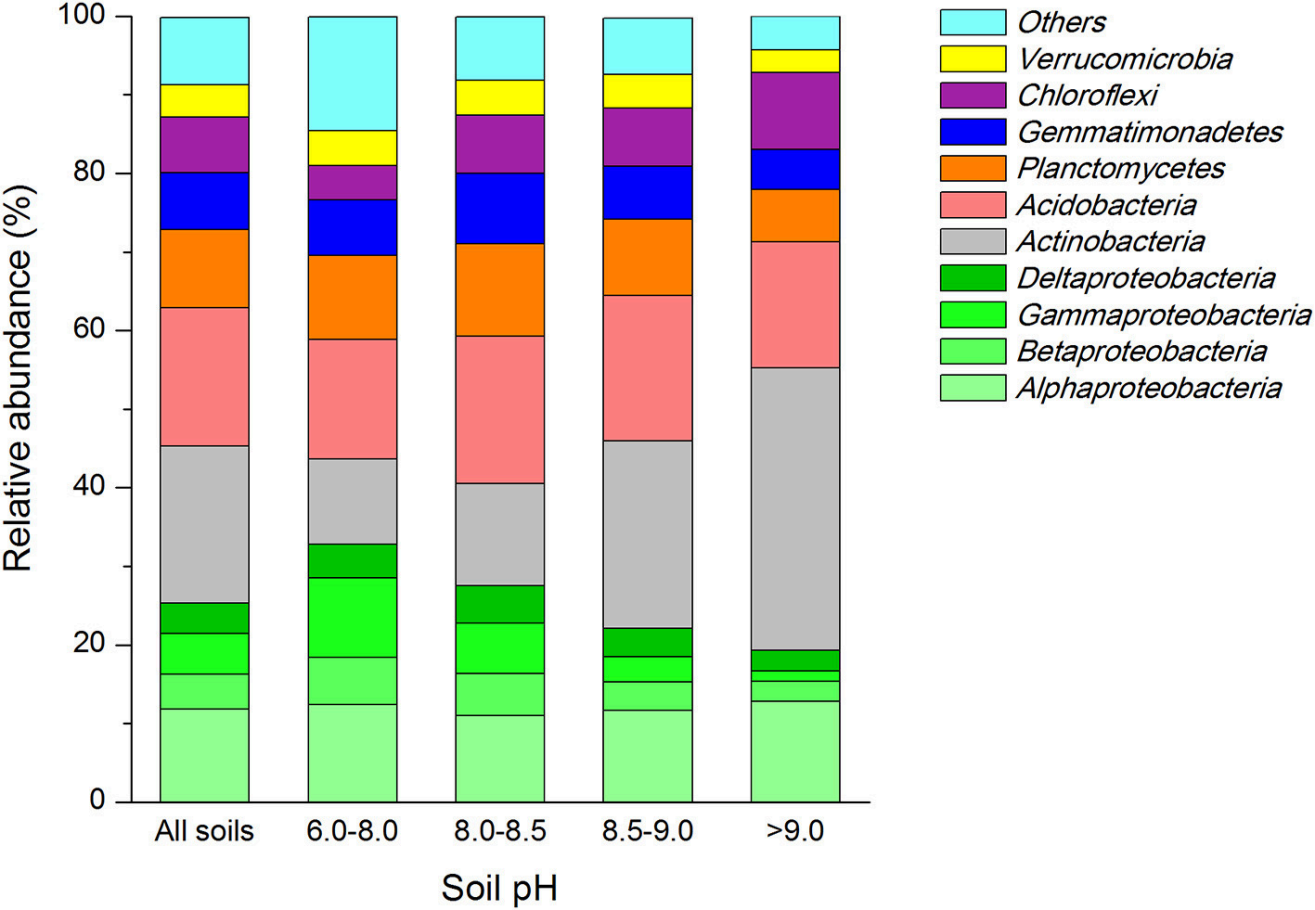
Relative abundances of the dominant bacterial groups across all soil samples and soils grouped into various pH gradients (values represent % of total non-redundant DNA sequences). To indicate their origin on the phylum level, colors were included, green indicate *Proteobacteria, Actinobacteria* gray, *Acidobacteria* are red, *Planctomycetes* are orange, *Gemmatimonadetes* are blue, purple refer to *Chloroflexi*, yellow represent *Verrucomicrobia*.

We obtained 14,11,001 high quality sequences from a total of 24 soil samples, and 49,729−72,997 sequences per sample (mean = 58,791). The read lengths of amplified V4 region of the 16S rRNA gene varied from 142 to 384 bp, at an average of 231 bp. When classified at the 97% similarity level, there were 74,093 different phylotypes in all soils. The average number of phylotypes in each sample was 3,087. The major bacterial taxa (relative abundance >5%) across all soil samples belonged to *Proteobacteria, Actinobacteria, Acidobacteria, Planctomycetes, Gemmatimonadetes, Chloroflexi*, and *Verrucomicrobia*. These groups were responsible for more than 90% of the total bacterial sequences obtained (Figure 1; Table S2). Groups of *Nitrospirae, Bacteroidetes, [Thermi], WS3, Firmicutes, Cyanobacteria, Armatimonadetes, and OD1* were less abundant (relative abundance >0.1% and <2.5%) but were still identified in most of the soil samples collected. On the class level, *Alphaproteobacteria, Acidobacteria-6* and *Thermoleophilia* were dominant with mean relative abundance of ~10%, followed by *Actinobacteria* (7%), *[Chloracidobacteria]* (6%) and *Planctomycetia* (5%). The classes of *Betaproteobacteria, Gammaproteobacteria* and *Deltaproteobacteria* were less abundant (~4%) as compared to *Alphaproteobacteria* (Table S3). At the order level, taxonomic classification showed that 170 orders were identified (Table S4). Among these orders *iii1-15, Rhizobiales, Actinomycetales, Solirubrobacterales* and *RB41* were most abundant (with a mean of ~7%). The orders of *Xanthomonadales, Gaiellales, Rhodospirillales, Pirellulales, Nitrospirales, [Chthoniobacterales], Gemmatales, WD2101, Myxococcales, Acidimicrobiales*, and *Burkholderiales* were also frequently detected, with a relative abundance ranging from 2 to 4%. Over 200 families were identified across soil samples (Table S5). Among them, the mean relative abundances of the top four families (~3%) were *Solirubrobacteraceae, Nocardioidaceae, Gaiellaceae*, and *Pirellulaceae*, the *[Chthoniobacteraceae], 0319-6A21, Bradyrhizobiaceae, Hyphomicrobiaceae, Gemmataceae, Rhodospirillaceae*, and *Xanthomonadaceae* were also observed in all soils, with mean relative abundance varing from 2 to 3%.

We found close positive relation of soil pH to the abundance of four dominant bacterial groups (Table 2). The relative abundance of *Betaproteobacteria* (*r* = −0.717, *P* < 0.001) and *Gammaproteo bacteria* (*r* = −0.544, *P* = 0.006) were significantly negatively correlated with soil pH, while *Actinobacteria* (*r* = 0.702, *P* < 0.001) and *Chloroflexi* (*r* = 0.561, *P* = 0.004) were significantly positively correlated. SOC, TN and total P (TP) contents correlated with the abundance of major dominant groups (Table 2). The abundance of *Alphaproteobacteria* only, showed significant relationship with TP (*r* = −0.568, *P* = 0.004) while the abundance of *Betaproteobacteria* exhibited high significant relationship with TN (*r* = 0.538, *P* = 0.007). Abundance of *Deltaproteobacteria* had a significant positive correlation with SOC (*r* = 0.561, *P* = 0.004) and TN (*r* = 0.581, *P* = 0.003) but no correlation with TP (*r* = 0.285, *P* = 0.176) was seen. A highly negative relationship between SOC (*r* = −0.663, *P* < 0.001), TN (*r* = −0.794, *P* < 0.001) and TP (*r* = −0.643, *P* < 0.001) was noted toward the abundance of actinobacterial members, while the abundance of *Acidobacteria* was only related with SOC contents (*r* = 0.468, *P* = 0.021) alone. The abundance of members of *Planctomyetes* and *Gemmatimonadetes* phylum was significantly positively correlated to the amounts of TOC (*r* = 0.417, *P* = 0.043 for *Planctomycetes*; and *r* = 0.506, *P* = 0.012 for *Gemmatinonadetes*) and TP (*r* = 0.533, *P* = 0.07 for *Planctomycetes*; and *r* = 0.814, *P* < 0.001 for *Gemmatinonadetes*). In contrast, the *Chloroflexi* members exhibited a significant negative correlation with SOC (*r* = −0.483, *P* = 0.017) and TN (*r* = −0.610, *P* = 0.002). Further, a significant relationship with the bacterial abundance and soil moisture only existed for bacterial groups belonging to *Chloroflexi* phylum (*r* = −0.487, *P* = 0.016).

**Table 2:** Pearson corelationship (*r*) and significance valuess (*P*) between realtive abundances of dominant soil bacterial groups and soil organic C, total N and P contents, and moisture.

**Taxon**	**pH**		**SOC**		**TN**		**TP**		**Moisture**	
	**r**	***P***	**r**	***P***	**r**	***P***	**r**	***P***	**r**	***P***
*Alphaproteobacteria*	−0.168	0.433	−0.255	0.229	0.008	0.972	−0.568^**^	0.004	−0.041	0.849
*Betaproteobacteria*	−0.717^**^	<0.001	0.238	0.263	0.538^**^	0.007	0.071	0.742	0.04	0.853
*Gammaproteobacteria*	−0.544^**^	0.006	0.087	0.687	0.373	0.072	0.207	0.331	0.273	0.197
*Deltaproteobacteria*	−0.351	0.093	0.561^**^	0.004	0.581^**^	0.003	0.285	0.176	0.127	0.554
*Actinobacteria*	0.702^**^	<0.001	−0.663^**^	<0.001	−0.794^**^	<0.001	−0.643^**^	0.001	−0.39	0.059
*Acidobacteria*	−0.001	0.997	0.468^*^	0.021	0.28	0.185	−0.044	0.836	0.11	0.609
*Planctomycetes*	−0.222	0.297	0.417^*^	0.043	0.387	0.062	0.533^**^	0.007	0.204	0.34
*Gemmatimonadetes*	−0.007	0.974	0.506^*^	0.012	0.271	0.201	0.814^**^	<0.001	−0.055	0.797
*Chloroflexi*	0.561^**^	0.004	−0.483^*^	0.017	−0.610^**^	0.002	−0.227	0.287	−0.487^*^	0.016
*Verrucomicrobia*	−0.318	0.129	0.261	0.218	0.255	0.23	−0.105	0.624	0.035	0.871

* P < 0. 05,

** *P < 0.01*.

### Bacterial community diversity

**Figure 2:**
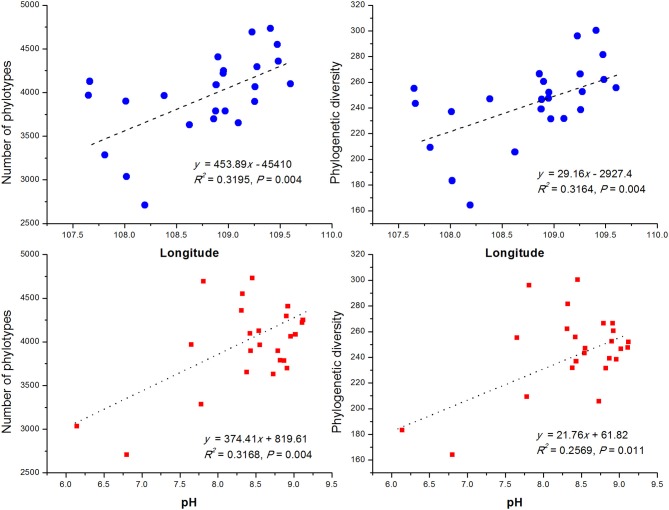
The relationship between longitude of sampling sites, soil pH and soil bacterial OTUs phylotype richness and phylogenetic diversity. The communities were randomly selected at the 45,000 sequences level.

In order to compare the soil bacterial community diversity across all landscape scales, a threshold of 45,000 sequences (per sample in the sequencing library) was used in order to minimize the survey effort. In all 24 soils, we observed a significant abundance in phylotypic richness ranging from 2,710 to 4,732 sequences mainly belonging to the phyla of *Proteobacteria, Actinobacteria, Acidobacteria, Planctomycetes, Gemmatimonadetes, Chloroflexi, and Verrucomicrobia* (Table 1; Figure 1). Moreover, a high phylogenetic diversity ranged from164 to 300 was also found in all soils (Table 1). The diversity of bacterial community increased with the increasing of geographic longitude as revealed by pairwise correlation analysis (Figure 2). Among the analyzed soil characteristics only soil pH was significantly positively corrected with both, phylotype richness (*r* = 0.563, *P* = 0.004) and the phylogenetic diversity (*r* = 0.508, *P* = 0.011; Figure 2). Soil TP exhibited positive relationship with phylotype richness (*r* = 0.095, *P* = 0.658) and the phylogenetic diversity (*r* = 0.102, *P* = 0.634) while SOC and TN were both positively related with both phylotype richness and diversity with no significance (data not shown).

### Bacterial community structure

**Figure 3:**
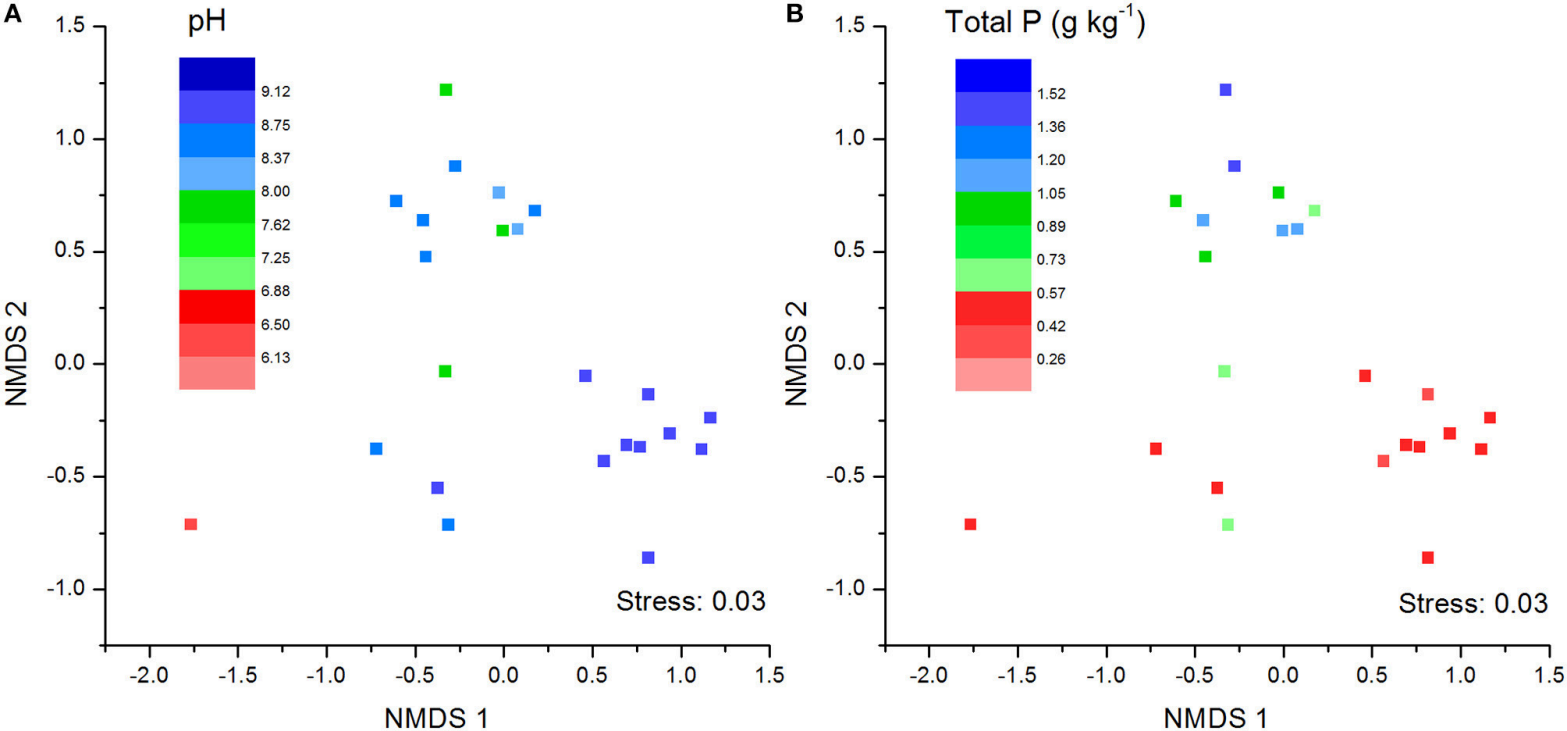
Soil bacterial community structure as indicated by unweighted no-metric multi-dimensional scaling plots (NMDS) of pairwise UniFrac community distance across sites. Sampling sites have been color-coded by pH values **(A)** and total phosphorus content **(B)**.

**Figure 4:**
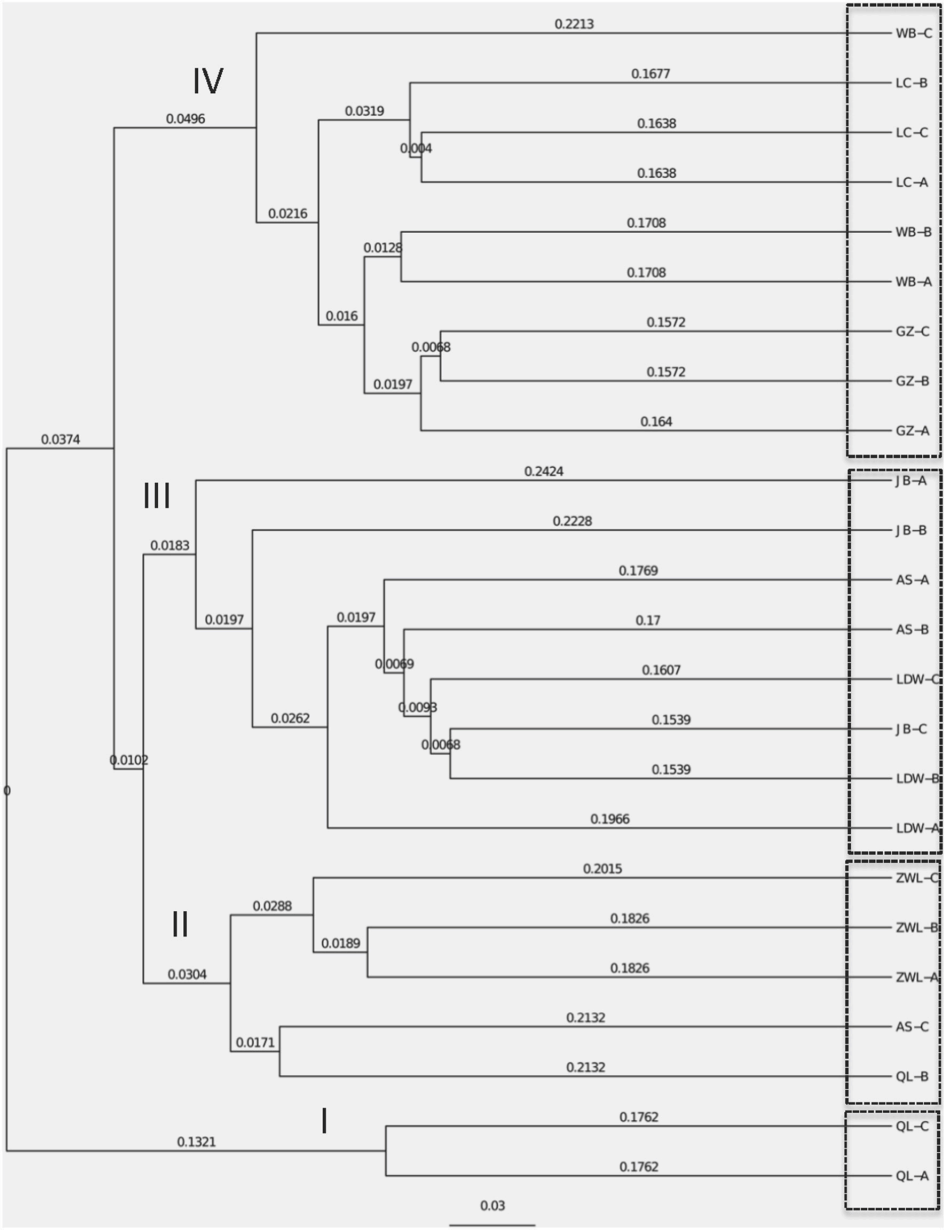
Cluster analysis of soil bacterial structure based on the dissimilarity matrix of the NMDS analysis. OL and ZWL represent locations from Qingling and Ziwuling mountain forests, respectively; LDW represents sites from grassland Liandaowan township; GZ, LC, and WB refer farming areas from three Loess tablelands–Guanzhong, Luanchuan, and Weibei, respectively. AS and JB from regions of forest steppes (Ansai county) and dessert steppes (Jingbian county). The codes of A, B, and C after the above-mentioned site abbreviations represent various biological replicates at landscape scales.

**Figure 5:**
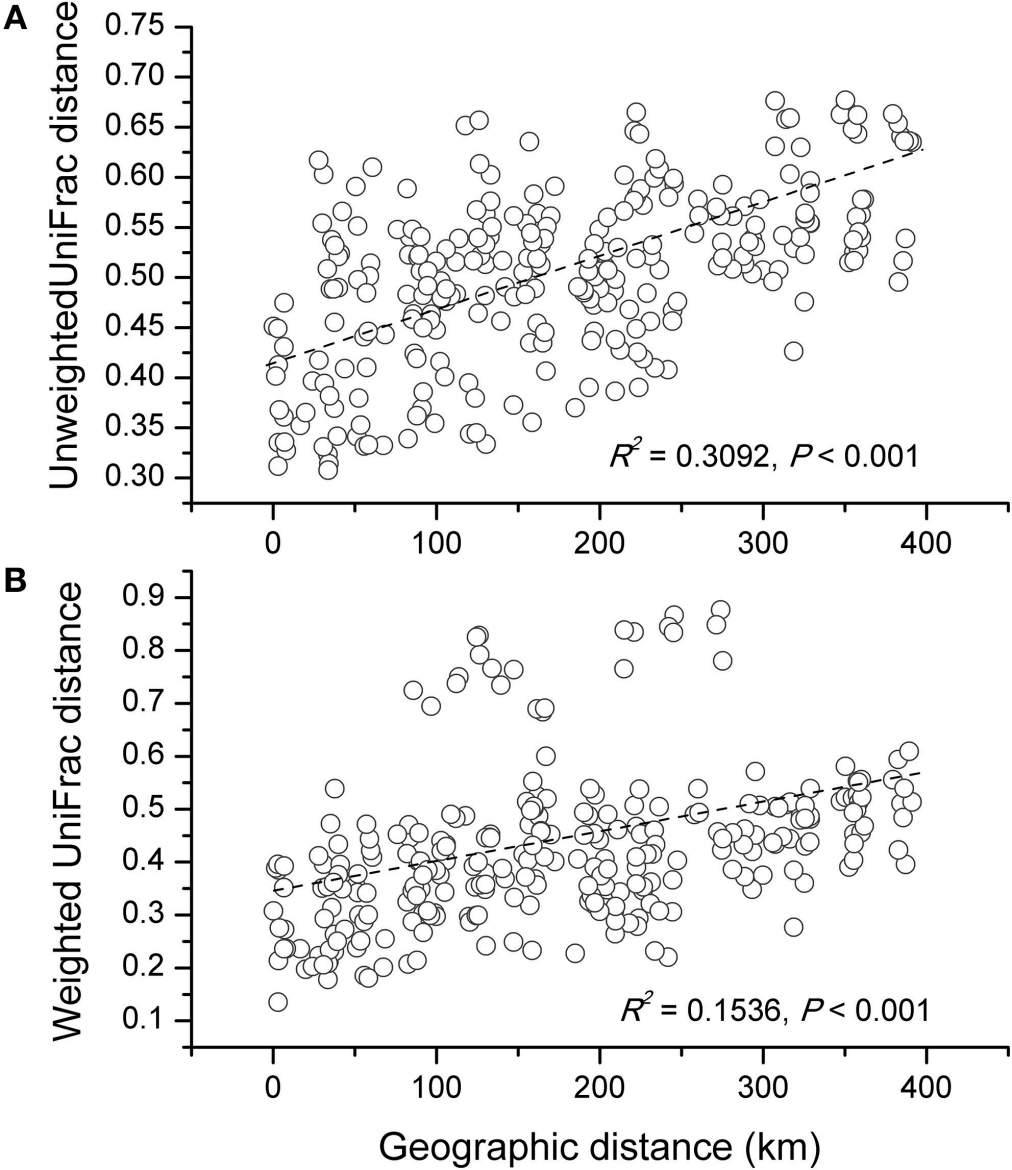
Correlationship between the unweighted **(A)** and weighted **(B)** UniFrac differences in soil microbial community structure and the distance of microbes-inhabiting geographic locations.

The NMDS plots of the unweighted and weighted pairwise UniFrac community distance ordinations clearly indicated that the bacterial community structure in the collected soil samples were strongly influenced by soil pH (Mantel test *r* = 0.514, *P* = 0.001; Figure 3A) and soil TP contents (Mantel test *r* = 0.367, *P* = 0.008; Figure 3B). The regression analysis between the NMDS scores, soil pH and TP contents indicated that soil pH was significantly related with NMDS1 (*r* = 0.833, *P* = 0.001) while soil TP had significantly higher linear relationship with NMDS2 (*r* = 0.842, *P* = 0.001; Table 3). This suggests that the variations in the bacterial community structure were firstly explained by soil pH along NMDS1 axis and then by soil TP contents along NMDS2 axis. This observation was also supported by CCA analysis of OTUs and soil parameters. CCA plots clearly demonstrate the strongest effects of soil pH and TP contents represented by two longest arrows directed along the X and Y axes, respectively (Figure S3) on distribution of bacterial OTUs belonging to different soil samples. Based on the NMDS dissimilarity matrix, the bacterial communities in 24 soil samples were classified into four major groups (Figure 4). Group I contains only two acidic soils, and the rest alkaline soils grouped clearly by TP contents. The lowest TP soils observed in Group III (0.26–0.53 g kg^−1^). Except for the ZWL-B soil samples (TP = 0.47 g kg^−1^), Group II had TP contents in a range of 0.54–0.59 g kg^−1^. Among all groups, Group IV had relatively highest TP contents (0.68–1.52 g kg^−1^). As the first grouping factor, soil pH showed significant positive correlations with geographic distance (*r* = 0.506, *P* = 0.012), and with microbial community (Table 2). Further, the unweighted and weighted UniFrac distances of the bacterial communities across the 24 soil samples were both significantly (*P* < 0.001) correlated with geographical distance (Figure 5) with higher R^2^ (goodness of Fit) values 0.3092 and 0.1536, respectively. Together, the results indicate a spatial pattern of soil bacterial structure along distinct landscapes on the Loess Plateau.

**Table 3:** Pearson correlation (*r*) and significance (*P*) between the unweighted NMDS scores and soil pH and total phosphorus (TP) content.

**Soil variables**	**Unweighted NMDS scores**			
	**NMDS 1**		**NMDS2**	
	***r***	***P***	***r***	***P***
pH	0.833^**^	<0.001	0.016	0.94
TP	−0.305	0.147	0.842^**^	<0.001

P < 0. 05,

** *P < 0.01*.

### Contributions of soil characteristics and geographic distance to bacterial community

**Figure 6:**
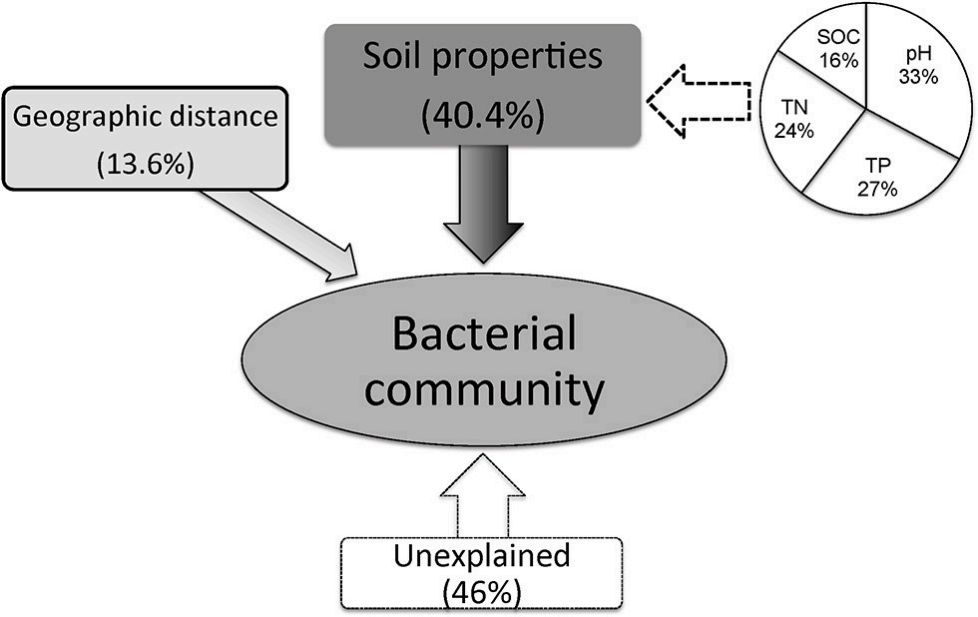
Variation partition analysis of the effects of soil properties and geographic distance on the phylogenetic structure of soil bacterial communities.

Variance partitioning analysis (VPA) was conducted to quantify the relative contributions of the geographic distance, soil characteristics and bacterial community structure. A subset of soil parameters (pH, TOC, TP, TN, and SOC) having significant correlations (*P* < 0.01) with microbial communities matrix was selected for CCA analysis. The combination of soil characteristics and geographic distances showed a significant correlation (*r* = 0.541, *P* < 0.001) with the soil bacterial community structure. The soil variables explained 54.0% while geographic distance explained only 13.6% of the variations in bacterial communities. Among the selected soil parameters, soil pH, TP, TN and SOC explained 33%, 27%, 24% and 16% of variations in bacterial community structure, respectively (Figure 6). Therefore, the intrinsic soil properties were more important than geographical distance for determining the distribution of bacterial communities in loess soils.

## Discussion

### Relative abundance of dominate bacterial communities

All of the loess soils analyzed were dominated by seven bacterial phyla (*Proteobacteria, Actinobacteria, Acidobacteria, Planctomycetes, Gemmatimonadetes, Chloroflexi*, and *Verrucomicrobia*). The relative average abundance of the top five major bacterial groups *Proteobacteria* (total 25%), *Actinobacteria* (20%)*, Acidobacteria* (17%)*, Planctomycetes* (10%), and *Gemmatimonadetes* (7%) found in this study were consistent with findings using 115 typical forest soil samples from an eastern Chinese region (Xia et al., 2016). In addition, the changes of the major soil bacterial groups among various land-use types were similar with our previous results where soils were collected from 15 sites associated with various types of vegetation (Zeng et al., 2016), that is, the relative abundance and prevalence of copiotrophic phylum *Proteobacteria* decreased in nutrient-poor and more alkaline soils while the abundance of oligotrophic *Acidobacteria* exhibited minor change (~20%; Figure 1). However, these groups were different from those observed in 88 soil samples across North and South American soils (Lauber et al., 2009) where the *Acidobacteria* was the most dominant phylum (averaged 32%) and the relative abundance of *Acidobacteria* obviously declined with increasing soil pH, whereas the abundance of *Proteobacteria* tended to be stable (~25%) in all soils. The relative abundance of *Gemmatimonadetes* and *Chloroflexi* (7%) was seven times higher than those taxa found in American soils (Lauber et al., 2009; <1%). In this study, a high relative abundance of *Beta-* and *Gamma-proteobacteria* (5%) was found; these phyla were reported rare in British soils by Griffiths et al. (2011). The differences in abundance of soil bacterial phyla in the Chinese Loess soils in comparison to other places of the world, suggest a differential pattern of bacterial dominance at the global scale. However, having known who persists where, a meta-analysis to reveal functional diversity of these bacterial communities across the globe would be of great potential in future research activities.

### Effect of soil pH

The unbiased role of soil pH in shifting and structuring soil bacterial community structure has been documented in numerous research activities across various soils in Britain (Griffiths et al., 2011), North and South America (Lauber et al., 2009), further in Arctic (Chu et al., 2010), arable (Rousk et al., 2010; Liu J. et al., 2014) as well as forest soils (Xia et al., 2016). Response of soil microbes to differential pH range had been studied extensively at various soils pH values ranging from acidic to neutral (Lauber et al., 2009; Chu et al., 2010; Griffiths et al., 2011; Xia et al., 2016) to hyper alkaline conditions where soil pH was as high as 10.3 (Xiong et al., 2012). In the present investigation, loess soils exhibited a pH range of 6.8 to 9.1 (Table 1). Similar to Xiong et al. (2012) we did observe a highly significant correlation of soil bacterial community structure and diversity (indicated by phylogenetic richness and diversity) with soil pH (Table 2; Figure 2), in addition to the marked variations in bacterial communities across the studied pH range of surveyed loess soils (Figure 3). Further it would be highly important to discriminate the dominant role of soil pH in shaping bacterial communities by exclusively studying its single effect or by incorporating an indirect function with other substantial environmental variables, such as soil nutrients and vegetation types (Lauber et al., 2009; Rousk et al., 2010; Liu J. et al., 2014). Based on this paucity, Liu J. et al. (2014) studied the direct effect of soil pH in changing bacterial communities in a single agricultural ecosystem where soil pH differed only by 2 units, but was accompanied by strong variations in soil organic carbon (SOC) and nitrogen contents. To this end, our results demonstrate that, although soil nutrients exhibited varying ranges of SOC-3.4 times, TN-7.4 times and TP-4.5 times across different ecosystems than soil pH ~ <2 times, soil pH still served as an overarching factor responsible for alterations in soil bacterial communities (Figures 2, 3). The influences of soil pH on the relative abundance of some bacterial taxa are similar/different from other studies. For instance, the relative abundance of *Acidobacteria* was shown to decrease at higher pH values (Rousk et al., 2010; Shen et al., 2013), while in our study it remained stable with no significant relationship (*P* > 0.05) toward observed pH, which can be ascribed to inconsistent responses of *Acidobacterial* subgroups to changing pH gradients (Liu J. et al., 2014). Further, it might also be important to note that pH alone is not the controlling parameter for this group in soils although it might be of strict importance in procedures where isolation of this group is the main aim. The abundance of *Chloroflexi* was significantly positively correlated with soil pH (*P* < 0.01, Table 2), similar results have also been reported in agricultural and forest soils in China as the optimum pH range required for growth by bacterial members belonging to *Chloroflexi* phylum is 5.5 to 8 as suggested by Hugenholtz and Stackebrandt (2004). *Actinobacteria* are widely distributed in soil with high sensitivity to low pH (Hopwood, 2007). However, in alkaline conditions, the relative abundance of *Actinobacteria* showed significantly positively relationship with pH both in our study (Table 2) as well as in Malaysian soils as studied by (Tripathi et al., 2012). The negative relationship between the abundance of *Proteobacteria* and soil pH have been previously observed in soils by Shen et al. (2013). However, differential responses of *Proteobacteria* to pH were observed at class levels, for examples, *Alpha-* and *Deltaproteobacteria* showed significant positive and negative relationships in alkaline sediment soils (Xiong et al., 2012), respectively. While in acidic forest soils, opposite significant relationships of these two classes were reported by Xia et al. (2016). Comparatively, in our study, we observed significant relationships between pH and *Beta-* and *Gammaproteobacteria* (*P* < 0.01, Table 2) but not for *Alpha-* and *Deltaproteobacteria*. These varying results keep the response of this group toward changing pH conditions unclear. Overall, the aforementioned findings indicate that the effect of soil pH on the distribution of bacterial taxa in the Loess Plateau soils was distinct from other soils, further suggesting the probability of other soil parameters playing crucial role in modulating bacterial communities in these soils.

### Effect of soil total P content

Significant correlations of SOC, TN and TP with most of the dominant phyla in our study were in line with the results of earlier investigations (Hollister et al., 2010; Griffiths et al., 2011; Liu J. et al., 2014; Xia et al., 2016). This is contrary to our expectations of uneven effects of C, N, and P on the bacterial community structures as the collected soils were from arid and semi-arid regions of the Loess Plateau, characterized by low TP, TN (Liu et al., 2013), and SOC (<0.7%, Table 1) contents. Our results of Non Metric Multi-Dimensional Scaling (NMDS) revealed that the variations of soil microbial communities along NMDS1 and NMDS2 were significantly related to changes in soil pH and TP contents, respectively (Figure 3). This signifies the importance of soil TP contents along with soil pH, over TN and SOC contents in structuring the soil bacterial communities of Loess Plateau. Although TP is closely related to SOC (*r* = 0.548, *P* < 0.01) and TN (*r* = 0.411, *P* = 0.04) contents in soils, we wanted to bring to attention the control of TP on soil bacterial communities which is rarely discussed in earlier investigations.

We observed the significant negative relationship of soil TP with dominant bacterial phyla: *Actinobacteria* and *Alphaproteobacteria* (Table 2), which is consistent with earlier findings of Liu D. et al. (2014) and Sul et al. (2013). Additionally, the significant positive correlation between soil TP and relative abundance of *Gemmatimonadetes* and *Planctomycetes* are in agreement with Xia et al. (2016). The various responses of these bacterial groups highlight their capabilities to better adapt to low nutrient soil environments of the Loess Plateau, due to their copiotrophic/oligotrophic life histories (Fierer et al., 2007).

### Bacterial biogeographic distribution and their determining factors

Contemporary environmental heterogeneity (contemporary factor) and historical contingencies (dispersal limitation as reflected by geographic distances) are considered to be major driving factors for variations in microbial diversity (Martiny et al., 2006; O'Malley, 2007). However, their relative importance is still not clearly discussed. For example, the historical factor of geographic distance was previously considered of less importance (Finlay, 2002; Chu et al., 2010), or regarded important only in presence of other soil characters shaping bacterial communities (Ge et al., 2008; Griffiths et al., 2011; Xiong et al., 2012; Ito et al., 2016) where it still received less attention than its share. In our work we observed classification of collected 24 soil samples into four groups. Except for two acidic soils in group I, the rest soils were alkaline in nature mainly because of the vegetation-belts imparting this characteristic feature to the soils in Group II, III, and IV; Figure 4, respectively, which further followed a south-west to north-east transect (Figure S2). Furthermore, a significant relationship was found between sampling locations separated by geographical distances and soil microbial community structure (Figure 5). Our results clearly indicate the unbiased influence of geographic distance on soil bacterial communities of Loess Plateau further exhibiting a vegetation belt-related geographic pattern of microbial community distribution. Similar to the findings of Liu J. et al. (2014) and Xia et al. (2016), geographic distance explained though low but 13.6% of the variations in bacterial community members in this region (Figure 6).

By comparing our results with other investigations conducted at various scales, we noticed that the importance of environmental variables (indicated on percent basis responsibility for microbial communities distribution) tend to increase with the decreasing of sampling scales. At a regional scale (~1,000 km), environmental variables only explained 21% of the variation in bacterial communities (Xia et al., 2016), however at a moderate scale of ~600 km, Liu J. et al. (2014) found the contribution of soil variables to microbial change was 38%. In this study, at a landscape scale (~400 km) soil properties together explained 41% (Figure 6) of variations while individual soil properties explained >15% of variations in bacterial community structure in contrast to geographic distance which explained only 13.6% (Figure 6). However, apart from the above-mentioned two factors, still 46% of the variations remain unexplained in this study (Figure 6). We think the unmeasured soil texture, which determines the extent and connectivity of microhabitats, should be an important factor in constraining richness and diversity of soil bacterial communities (Chau et al., 2011). Apart from soil texture, other factors, such as soil aggregate structure, microbial biomass and aboveground vegetation may also contribute to unexplained variation in bacterial communities (Hansel et al., 2008; Thomson et al., 2010; Tang et al., 2011; Liu D. et al., 2014; Fu et al., 2015; Delgado-Baquerizo et al., 2017). Therefore, in order to explain changes related to the diversity of widely distributed soil bacteria, detailed investigations covering comprehensive soil properties are required. In terms of microbial biogeography, many studies have addressed the spatial scaling and distribution patterns of environmental microorganisms (Martiny et al., 2006; Green et al., 2008). Although, Kuang et al. (2013) reported weak contributions of spatial isolation represented by gradients of latitude and longitude to explain microbial diversity, it is still an improvement compared to earlier studies that described no latitudinal diversity gradient at all for soil microbial communities (Fierer and Jackson, 2006; Chu et al., 2010). However, it must be noted that the samples collected in these studies were either from extreme environments or remote habitats comprising diverse soil types; thus, potential microbial latitudinal/longitudinal diversity pattern may have been masked by other strong heterogeneous factors responsible for hosting differential microbial communities. In our study, we investigated only the loess soils across a moderate landscape scale of Chinese Loess Plateau. Soil bacterial diversity (as estimated by phylotype richness or phylogenetic diversity) showed positive but no significant relationship with latitudinal gradients (*r* = 0.31, *P* > 0.05). Contrastingly, bacterial diversity did show a significant positive relationship (revealed by pairwise correlation analysis) with longitudes of the sampling locations (Figure 2). This observations strongly suggests longitudinal pattern of bacterial diversity existing in Loess Plateau soils, which can be attributed to a clear vegetation belt distribution along the investigated longitude range (107°39′-109°15′; Figure S2). Similarly, Staddon et al. (1998) also found that in a south-east to north-west transect of soil sampling locations, both latitude and longitude showed correlation to soil microbial diversity.

## Conclusions

This study is an attempt to investigate biogeographic distribution patterns of bacteria communities across Loess Plateau soils. We report that bacterial community structure in these soils is strongly manipulated by soil pH and TP contents. The phylotype richness and phylogenetic diversity are exclusively predicted by both soil pH and longitude. Our results clearly indicate that endemic soil properties are the dominating factors in shaping the bacterial communities and equally responsible for their variations. In addition, geographical distance was also a critical factor contributing in variations of bacteria communities across a landscape scale (about 400 km). Since soil microorganisms play a significant role in many ecosystem processes, cataloging their community structure and changes would help to better predict landscape-scale responses to environmental changes, such as soil erosion and transformations. Further prospects of the work include understanding diversity patterns of another major group of soil microorganisms represented by fungal communities.

## Author contributions

DL is responsible for the manuscript writing, YY extracted DNA of all soil samples, SA designed the whole experiment, HW contributed to data analysis and software application, YW is respondsible for the sequencing data analysis.

### Conflict of interest statement

The authors declare that the research was conducted in the absence of any commercial or financial relationships that could be construed as a potential conflict of interest.
