# Supplementary material for: The Biogeographical Distribution of Soil Bacterial Communities in the Loess Plateau as Revealed by High-Throughput Sequencing
Source: Front Microbiol. 2018 Oct 18;9:2456. doi: 10.3389/fmicb.2018.02456 (PMC6200921; doi:10.3389/fmicb.2018.02456)
Supplement: Supplementary file 1 [file Table_1.DOCX]

| **Table S1** Pairwise geographic distance (km) matrix | | | | | | | | | | | | | | | | | | | | | | | |
| --- | --- | --- | --- | --- | --- | --- | --- | --- | --- | --- | --- | --- | --- | --- | --- | --- | --- | --- | --- | --- | --- | --- | --- |
|  | QL-A | QL-B | QL-C | GZ-A | GZ-B | GZ-C | LC-A | LC-B | LC-C | ZWL-A | ZWL-B | ZWL-C | LDW-A | LDW-B | LDW-C | AS-A | AS-B | AS-C | WB-A | WB-B | WB-C | JB-A | JB-B |
| QL-A | 0.0 |  |  |  |  |  |  |  |  |  |  |  |  |  |  |  |  |  |  |  |  |  |  |
| QL-B | 50.6 |  |  |  |  |  |  |  |  |  |  |  |  |  |  |  |  |  |  |  |  |  |  |
| QL-C | 16.6 | 36.0 |  |  |  |  |  |  |  |  |  |  |  |  |  |  |  |  |  |  |  |  |  |
| GZ-A | 60.8 | 28.1 | 51.9 |  |  |  |  |  |  |  |  |  |  |  |  |  |  |  |  |  |  |  |  |
| GZ-B | 30.9 | 37.6 | 30.1 | 33.6 |  |  |  |  |  |  |  |  |  |  |  |  |  |  |  |  |  |  |  |
| GZ-C | 27.9 | 68.6 | 42.3 | 67.5 | 34.2 |  |  |  |  |  |  |  |  |  |  |  |  |  |  |  |  |  |  |
| LC-A | 221.1 | 244.7 | 233.3 | 223.9 | 209.5 | 193.4 |  |  |  |  |  |  |  |  |  |  |  |  |  |  |  |  |  |
| LC-B | 224.3 | 247.6 | 236.4 | 226.6 | 212.5 | 196.6 | 3.4 |  |  |  |  |  |  |  |  |  |  |  |  |  |  |  |  |
| LC-C | 222.4 | 244.4 | 234.2 | 223.0 | 209.8 | 194.9 | 8.0 | 7.1 |  |  |  |  |  |  |  |  |  |  |  |  |  |  |  |
| ZWL-A | 226.5 | 241.8 | 236.5 | 218.1 | 209.7 | 200.0 | 41.2 | 40.0 | 33.2 |  |  |  |  |  |  |  |  |  |  |  |  |  |  |
| ZWL-B | 222.5 | 233.8 | 231.3 | 208.9 | 203.5 | 197.0 | 59.7 | 59.0 | 52.0 | 19.9 |  |  |  |  |  |  |  |  |  |  |  |  |  |
| ZWL-C | 224.9 | 230.9 | 232.2 | 204.8 | 203.2 | 200.8 | 83.7 | 83.1 | 76.1 | 43.9 | 24.1 |  |  |  |  |  |  |  |  |  |  |  |  |
| LDW-A | 350.4 | 355.6 | 357.8 | 328.9 | 328.8 | 325.7 | 161.9 | 159.0 | 155.3 | 132.7 | 129.9 | 125.6 |  |  |  |  |  |  |  |  |  |  |  |
| LDW-B | 347.4 | 352.5 | 354.7 | 325.7 | 325.7 | 322.8 | 159.9 | 157.0 | 153.2 | 130.2 | 127.1 | 122.6 | 3.2 |  |  |  |  |  |  |  |  |  |  |
| LDW-C | 350.4 | 355.4 | 357.7 | 328.6 | 328.7 | 325.8 | 162.7 | 159.7 | 156.0 | 133.2 | 130.1 | 125.5 | 1.6 | 3.0 |  |  |  |  |  |  |  |  |  |
| AS-A | 314.0 | 325.2 | 323.1 | 299.9 | 295.3 | 288.0 | 112.0 | 108.9 | 106.0 | 88.7 | 91.8 | 96.4 | 52.5 | 51.2 | 53.6 |  |  |  |  |  |  |  |  |
| AS-B | 307.4 | 318.8 | 316.5 | 293.5 | 288.7 | 281.3 | 105.9 | 102.8 | 99.8 | 82.1 | 85.3 | 90.4 | 57.4 | 55.9 | 58.4 | 6.6 |  |  |  |  |  |  |  |
| AS-C | 307.3 | 318.7 | 316.4 | 293.4 | 288.7 | 281.3 | 106.0 | 102.9 | 99.9 | 82.1 | 85.2 | 90.2 | 57.2 | 55.7 | 58.2 | 6.7 | 0.3 |  |  |  |  |  |  |
| WB-A | 117.7 | 157.2 | 133.2 | 147.2 | 119.7 | 91.1 | 120.3 | 123.7 | 124.5 | 142.1 | 147.6 | 161.6 | 274.8 | 272.3 | 275.2 | 228.9 | 222.4 | 222.4 |  |  |  |  |  |
| WB-B | 156.9 | 196.2 | 172.5 | 185.0 | 158.6 | 130.4 | 99.1 | 102.3 | 105.1 | 129.9 | 140.5 | 159.0 | 260.2 | 258.0 | 260.8 | 211.0 | 204.8 | 204.9 | 39.3 |  |  |  |  |
| WB-C | 126.1 | 113.8 | 126.6 | 85.8 | 96.9 | 112.2 | 165.1 | 167.1 | 161.7 | 147.4 | 134.0 | 124.7 | 245.5 | 242.3 | 245.1 | 221.0 | 215.0 | 214.8 | 139.5 | 166.4 |  |  |  |
| JB-A | 384.5 | 387.2 | **391.2** | 360.0 | 361.9 | 360.4 | 199.7 | 196.7 | 193.1 | 170.0 | 166.1 | 159.6 | 37.8 | 40.0 | 37.1 | 89.4 | 94.7 | 94.6 | 312.0 | 297.9 | 275.3 |  |  |
| JB-B | 382.6 | 385.7 | 389.5 | 358.5 | 360.2 | 358.5 | 197.1 | 194.1 | 190.5 | 167.6 | 163.9 | 157.8 | 35.2 | 37.5 | 34.6 | 86.6 | 92.0 | 91.8 | 309.7 | 295.3 | 274.0 | 2.9 |  |
| JB-C | 379.5 | 382.9 | 386.4 | 355.8 | 357.2 | 355.3 | 193.2 | 190.3 | 186.7 | 164.0 | 160.5 | 154.6 | 31.4 | 33.8 | 30.8 | 82.7 | 88.1 | 87.9 | 306.0 | 291.6 | 271.4 | 6.7 | 3.9 |

**Table S2** Relative average abundances of bacterial groups across all soil samples and soils classified into different pH categories (values represent % of total DNA sequences).

| Bacterial taxon | All | pH  6.0-8.0 | pH  8.0-8.5 | pH  8.5-9.0 | pH  >9.0 |
| --- | --- | --- | --- | --- | --- |
| *Alphaproteobacteria* | 11.83 | 13.52 | 10.94 | 11.42 | 12.38 |
| *Betaproteobacteria* | 4.47 | 6.51 | 5.39 | 3.61 | 2.47 |
| *Gammaproteobacteria* | 5.21 | 11.00 | 6.40 | 3.12 | 1.23 |
| *Deltaproteobacteria* | 3.92 | 4.62 | 4.76 | 3.52 | 2.60 |
| *Actinobacteria* | 20.04 | 11.85 | 13.03 | 23.40 | 34.74 |
| *Acidobacteria* | 17.65 | 16.52 | 18.66 | 18.23 | 15.46 |
| *Planctomycetes* | 10.07 | 11.61 | 11.75 | 9.47 | 6.48 |
| *Gemmatimonadetes* | 7.21 | 7.73 | 9.03 | 6.60 | 4.95 |
| *Chloroflexi* | 7.08 | 4.69 | 7.31 | 7.31 | 9.40 |
| *Verrucomicrobia* | 4.21 | 4.87 | 4.47 | 4.19 | 2.83 |
| *Nitrospirae* | 2.58 | 4.10 | 2.80 | 2.20 | 1.14 |
| *Bacteroidetes* | 2.32 | 0.66 | 1.50 | 3.68 | 1.89 |
| *[Thermi]* | 0.93 | 0.06 | 1.21 | 1.35 | 0.28 |
| *WS3* | 0.78 | 1.50 | 0.98 | 0.48 | 0.27 |
| *Firmicutes* | 0.91 | 0.34 | 0.85 | 0.59 | 2.89 |
| *Cyanobacteria* | 0.31 | 0.06 | 0.22 | 0.37 | 0.66 |
| *Armatimonadetes* | 0.28 | 0.30 | 0.25 | 0.30 | 0.24 |
| *OD1* | 0.14 | 0.05 | 0.36 | 0.06 | 0.07 |
| *BRC1* | 0.07 | 0.04 | 0.09 | 0.09 | 0.03 |
