# Supplementary material for: The Biogeographical Distribution of Soil Bacterial Communities in the Loess Plateau as Revealed by High-Throughput Sequencing
Source: Front Microbiol. 2018 Oct 18;9:2456. doi: 10.3389/fmicb.2018.02456 (PMC6200921; doi:10.3389/fmicb.2018.02456)
Supplement: Supplementary file 3 [file Data_Sheet_1.docx]

**Fig. S1** The typical landscapes of the studied areas on the Loess Plateau.


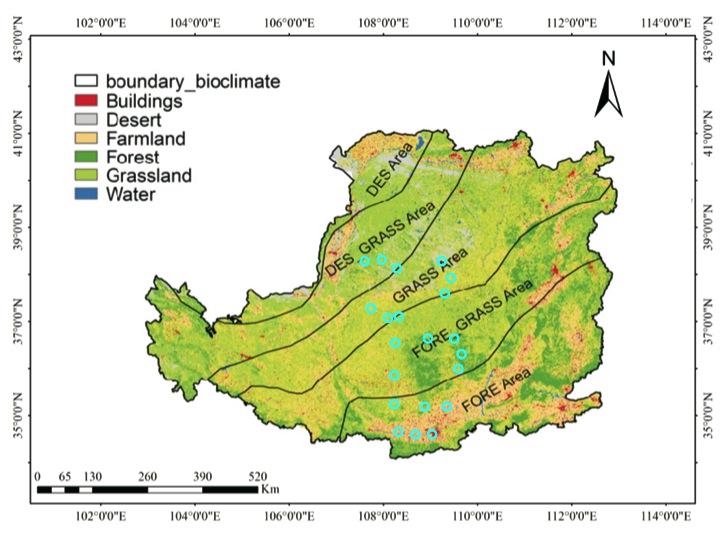


**Fig. S2** Vegetation-belt distribution from southwest to northeast transect.

**Fig. S3.** Canonical correlation analysis (CCA) of soil bacterial structure.
